# Unveiling the Phenotypic Spectrum of Miller Syndrome: A Systematic Review

**DOI:** 10.1097/SCS.0000000000011501

**Published:** 2025-05-19

**Authors:** Victor L. van Roey, Saranda Ombashi, Idilay Kaymaz, Marieke F. van Dooren, Anne Goverde, Eppo B. Wolvius, Irene M.J. Mathijssen, Sarah L. Versnel

**Affiliations:** * European Reference Network for rare and/or complex craniofacial anomalies and ear, nose, and throat disorders; † Department of Plastic and Reconstructive Surgery, Erasmus University Medical Centre; ‡ Department of Oral and Maxillofacial Surgery, Erasmus University Medical Centre; § Department of Clinical Genetics, Erasmus University Medical Centre, Rotterdam, The Netherlands

**Keywords:** Acrofacial dysostosis, Genee-Wiedemann syndrome, Miller syndrome, POADS, postaxial acrodysostosis, systematic review, Wildervanck-Smith syndrome

## Abstract

Miller syndrome is an extremely rare condition in the group of facial dysostosis syndromes. These syndromes have great phenotypic overlap and variability, even within families. To facilitate the differentiation of Miller syndrome from related facial dysostosis syndromes, such as Treacher Collins and Nager syndrome, this study aims to provide an overview of the phenotypic spectrum of the syndrome. A systematic literature search of Embase, MEDLINE/PubMed, Web of Science, and CINAHL was conducted until November 2024. Case reports and case series of patients with a clinical or genetic diagnosis of Miller syndrome in all languages were included. The quality of the included reports was assessed using the Joanna Briggs Institute critical appraisal tool and the Fichas de Lectura Critica 3.0 web application. In total, 44 cases of Miller syndrome were found, with only 18.2% having genetic confirmation. Craniofacial anomalies were prominent, including midface hypoplasia (72.7%) and micrognathia (75.0%), orofacial clefts (77.3%), eyelid anomalies (70.5%), and external ear anomalies (63.6%). Limb anomalies were present in all cases, primarily involving the hands (95.5%), forearms (52.3%), and feet (90.9%). Anomalies in other extracraniofacial tracts were also reported. Despite limitations, including limited genetic confirmation and reliance on literature, this study provides valuable insights into the phenotypic spectrum of Miller syndrome. Efforts for genetic confirmation, international collaboration, and comprehensive reporting are essential to advance research and care for rare conditions like Miller syndrome. Therefore, a detailed checklist for phenotypic evaluation in Miller syndrome cases is provided in this study.

Miller syndrome, also referred to as postaxial acrofacial dysostosis syndrome (POADS), Genée-Wiedemann syndrome, and Wildervanck-Smith syndrome (OMIM #263750), is a rare condition within the group of facial dysostosis syndromes, characterized by craniofacial and limb anomalies. Although the exact prevalence of Miller syndrome is currently still unknown, it is estimated to affect less than one in a million newborns.^1^ The condition follows an autosomal recessive inheritance pattern and is attributed to biallelic pathogenic variants in the dihydroorotate dehydrogenase (*DHODH*) gene, a discovery made around 2010.^2,3^ This gene, located at chromosome 16q22.2, encodes an integral protein involved in the de novo pyrimidine biosynthesis pathway within mitochondria.^4^ The precise role of DHODH in embryonal development, however, remains unknown at this point.

Phenotypic characteristics in Miller syndrome closely resemble those found in other facial dysostosis syndromes, such as Treacher-Collins syndrome, which typically lacks limb anomalies,^5^ and Nager syndrome, where preaxial limb anomalies are generally more prominent.^6,7^ These syndromes exhibit great phenotypic variability, even within families.^8–11^ The phenotypic overlap and variability, together with their rarity, can pose challenges in distinguishing between facial dysostosis syndromes for (targeted) genetic testing, accurately informing clinicians and parents in case of prenatal diagnosis, and genetic counseling for family planning. Furthermore, although the treatment of facial dysostosis syndromes is broadly similar and personalized to the patient, it is important to rule out potential differences between the syndromes that may require additional attention, especially given their diverse aetiology.^12^

So far, limited research has been done on Miller syndrome patients, with the literature primarily consisting of case reports and case series. The largest study to date involved a review of ten cases of Miller syndrome by Donnai et al^13^ in 1987. Since then, several more cases have been described. To assist clinicians in recognizing and diagnosing this condition in its diverse presentations and to be able to better inform patients or their parents on the diagnosis, this study provides an updated overview of the phenotypic spectrum of Miller syndrome. In addition, to improve the reporting of future cases, this study offers a checklist for phenotypic evaluation. Finally, a comparison is made with the characteristics observed in Treacher-Collins and Nager syndrome to improve differentiation between these syndromes.

## METHODS

The current study was reported in accordance with the Preferred Reporting Items for Systematic reviews and Meta-Analyses (PRISMA) 2020 guidelines^14^ (Supplemental Digital File 1, Supplemental Digital Content 1, http://links.lww.com/SCS/H872).

### Literature Search and Eligibility Criteria

The Embase, MEDLINE (PubMed), Web of Science, and CINAHL databases were systematically searched (search strings in Supplemental Digital File 2, Supplemental Digital Content 2, http://links.lww.com/SCS/H873) for articles published from inception to November 2024, without language restrictions. The title and abstract of all found articles were independently screened by 2 reviewers using our eligibility criteria. The full texts of all remaining articles were subsequently screened using the same criteria and by the same reviewers. A snowball search was additionally performed by searching the citation lists of the included articles. Differences in the selected articles were resolved through discussion. Reference manager program EndNote 20 was used for the entire literature search, according to the methods of Bramer et al.^15^

All original case reports or case series on liveborn human patients with a clinical and/or genetic diagnosis of Miller syndrome were eligible for inclusion. Case reports in which the authors could not confirm Miller syndrome, either clinically or genetically, were excluded. Articles that did not report separate results for patients with Miller syndrome were also excluded, and studies exclusively investigating the aetiology and/or pathogenesis.

### Data Extraction and Quality Assessment

From the included articles, the following data were collected independently by 2 reviewers: author(s), year of publication, geographical location(s), the patient’s sex, and the basis of their diagnosis (clinical or genetic). All reported phenotypical characteristics were additionally collected and categorized according to the main craniofacial features (ie, ears, eyes and eyelids, nose, cheekbones, mouth, and jaws) or extracraniofacial tracts (ie, limb, vertebral, cardiac, central nervous system, gastrointestinal, renal, pulmonary, and genital anomalies). Anomalies that could not be categorized into these groups were categorized as “others”. Anomalies reported in more than 3 cases were included in the results, whereas less frequently reported anomalies are detailed in the checklist provided in Supplemental Digital File 3 (Supplemental Digital Content 3, http://links.lww.com/SCS/H874).

The quality of each included study was subsequently evaluated independently by 2 reviewers, using the Joanna Briggs Institute critical appraisal tool for case reports^16^ and the Fichas de Lectura Critica 3.0 web application^17^ for case series. Accordingly, all articles were categorized as low, average, or high quality.

## RESULTS

**FIGURE 1:**
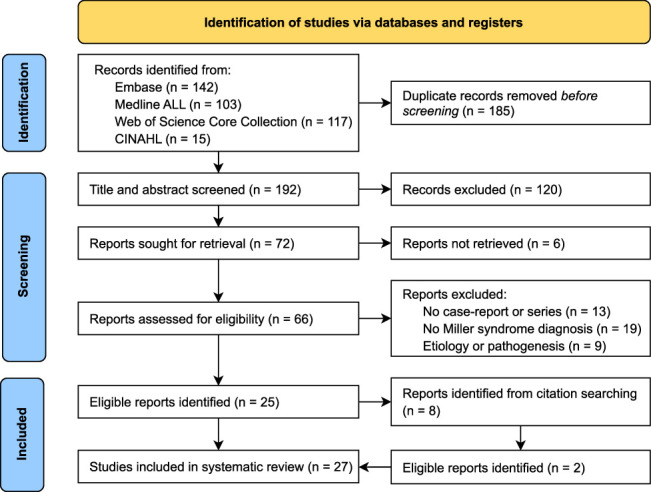
Flow chart of the article selection process.

Figure 1 shows the article selection process for this study. After deduplication and screening the remaining 193 articles, 25 met the eligibility criteria. In addition, 2 more articles were found in the snowball search. In total, these 27 articles provided information on 44 cases of Miller syndrome, representing 35 different families (Supplemental Digital Table 1, Supplemental Digital Content 4, http://links.lww.com/SCS/H875).^8,13,18–42^ Genetic confirmation of the diagnosis was available for 8 cases (18.2%) in the literature. A total of 19 centers with expertise in facial dysostosis syndromes from ten countries, along with 3 patient associations, were contacted to identify new cases of Miller syndrome. Two previously unreported cases were identified, both of whom did not provide consent for inclusion in the current study.

Geographically, the majority of the included cases originated from Europe (N=25, 56.8%), followed by North America (N=9, 20.5%), Oceania (N=4, 9.1%), South America (N=3, 6.8%), Asia (N=2, 4.5%), and Africa (N=1, 2.3%). In terms of sex distribution, slightly more cases of males (N=25, 56.8%) were reported than females (N=18, 40.9%). Most of the cases were born full-term (N=26, 59.1%), with some cases having unavailable data (NA: N=13, 29.5%). The reported average birth weight was 3.21 (SD 0.65) kg for 29 cases (NA: N=15, 34.1%), and the average length at birth was 49.2 (SD 4.67) cm for 19 cases (NA: N=25, 56.8%), both within the normal range. Three cases (6.7%) did not survive beyond 2 weeks after birth; respiratory insufficiency was the confirmed cause of death in 2 cases and was also suggested in the third.

### Summary of Craniofacial Characteristics

A broad spectrum of craniofacial anomalies was observed, as detailed in Supplemental Digital Table 2 (Supplemental Digital Content 5, http://links.lww.com/SCS/H875). Hypoplasia of craniofacial structures was the most prevalent (N=39, 88.6%), including micrognathia (N=33, 75.0%) and various degrees of midface hypoplasia (N=32, 72.7%). The majority of cases were also reported to have an orofacial cleft (N=34, 77.3%). Among these, 5 cases (11.4%) had a cleft lip and palate, whereas 29 cases (65.9%) presented with an isolated cleft palate. Different degrees of cleft palate were reported, including complete cleft palate (N=19, 43.2%), cleft soft palate only (N=8, 18.2%), or submucous cleft palate (N=2, 4.5%).

Eyelid anomalies were also prevalent, affecting at least 31 cases (70.5%). Slanting palpebral fissures were reported the most (N=19, 43.2%), both up-slanting (N=6, 13.6%) and down-slanting (N=13, 29.5%). Other frequently reported eyelid anomalies included ectropion (N=14, 31.8%), blepharophimosis (N=8, 18.2%), absent or scarce (medial) lower eyelashes (N=8, 18.2%), and colobomas of the upper or lower eyelids (N=9, 20.5%).

External ear anomalies were observed in 28 cases (63.6%). Cupped ears (N=20, 45.5%) and low-set ears (N=13, 29.5%) were the most frequently reported anomalies. Middle ear anomalies seemed less prevalent and were only described in 4 cases (9.1%).^23,24,31^ Few studies reported findings on hearing, but in those that did, hearing loss was reported in 8 cases (18.2%), and normal hearing was observed in 10 patients (22.7%). Six cases (13.6%) were further specified as having at least bilateral conductive hearing loss.

### Summary of Extracraniofacial Characteristics

Extracraniofacial anomalies were also frequently reported, with limb anomalies being a prominent feature in all patients. These limb anomalies primarily involved the hands (N=42, 95.5%), forearms (N=23, 52.3%), and feet (N=40, 90.9%). In both the hands and the feet, the most commonly reported anomalies were absent or malformed 5^th^ ray(s) (N=39, 88.6%; N=36, 81.8%, respectively), along with syndactyly of nonspecific rays (N=11, 25.0%; N=8, 18.2%, respectively). In addition, thumb absence or malformation was frequently described (N=17, 38.6%), as well as camptodactyly (N=7, 15.9%), a single palmar crease(s) (N=5, 11.4%), and carpal fusions (N=4, 9.1%). In general, anomalies of the forearms were poorly defined and typically involved varying degrees of ulna and/or radius hypoplasia (N=23, 52.3%). Radio-ulnar synostosis was also reported in 7 cases (15.9%).

Several cases also presented with anomalies affecting extracraniofacial tracts other than the limbs. Vertebral anomalies were the most common (N=12, 27.3%), such as pectus excavatum (N=10, 22.7%) and scoliosis (N=3, 6.8%). Cardiac anomalies were documented in 7 cases (15.9%), encompassing ventricular septal defects (N=4, 9.1%), patent ductus arteriosus (N=2, 4.5%), atrial septal defects (N=1, 2.3%), and tricuspid valve insufficiency (N=1, 2.3%). Genital anomalies exclusively affected males (N=7, 15.9%), and included cryptorchidism (N=6, 24.0%) and small or micro-penis (N=4, 16.0%). Gastrointestinal (N=4, 9.1%), central nervous system (N=4, 9.1%), and renal (N=2, 4.5%) anomalies were less frequently reported, with each anomaly occurring only once.

Normal cognitive development was confirmed in at least 24 cases (54.5%). Abnormal cognitive development was reported in 5 cases (11.4%) with at least one case attributed to perinatal hypoxia and microcephaly.^26^ The 4 remaining cases had mild delays in the development of speech or the achievement of developmental milestones.

### Genetically Confirmed Cases

In total, 8 cases with genetically confirmed Miller syndrome were identified. These patients were all from separate families. In total, these patients had 12 unique variants in the *DHODH*-gene (Supplemental Digital Table 3, Supplemental Digital Content 6, http://links.lww.com/SCS/H875); 11 missense variants and one frameshift variant. Two variants were reported in more than one patient (Arg135Cys in 5 patients and Arg346Trp in 2 cases). In most cases, the genetic diagnosis was obtained through direct Sanger sequencing of the *DHODH*-gene (N=6, 75.0%), followed by targeted next-generation sequencing (n=2, 25.0%).

### Comparison with Treacher-Collins and Nager

Supplemental Digital Table 4 (Supplemental Digital Content 7, http://links.lww.com/SCS/H875) summarizes phenotypic characteristics across genetically confirmed cases of Treacher-Collins,^43–46^ Nager,^6,7^ and Miller syndrome. High prevalences of external ear anomalies, eyelid anomalies, midface hypoplasia, and micrognathia were consistently reported across all 3 syndromes. However, specific features and their frequencies differed.

Although external ear anomalies were common in all 3 syndromes, Miller syndrome typically presented with less severe cupped-ear deformities rather than microtia, which is frequently seen in Treacher-Collins and Nager syndrome. In line with this, hearing loss was reported in 37.5% of genetically confirmed Miller syndrome cases, compared with 91.3% in Treacher-Collins and 71.4% in Nager syndrome cases. Lower eyelid colobomas were common in patients with Treacher-Collins syndrome (59.8%) but were less frequently reported in Miller syndrome (22.2%) and absent in Nager syndrome cases. Orofacial clefts were highly prevalent in patients with Miller or Nager syndrome (75.0% and 77.8%, respectively) but were much less common in Treacher-Collins syndrome (25.3%).

Extracraniofacial anomalies were notably more frequent in Nager and Miller syndrome compared with Treacher-Collins syndrome. Limb anomalies were universally present in Nager and Miller syndrome patients, whereas rare in Treacher-Collins syndrome. In patients with Miller syndrome, these primarily involved absent or malformed fifth rays, though thumb anomalies were occasionally reported (12.5%). Thumb anomalies were a consistent feature of Nager syndrome, but the occurrence of fifth-ray anomalies in Nager syndrome remains uncertain. In addition, vertebral, cardiac, and gastrointestinal anomalies were particularly more common in Miller syndrome than in patients with Treacher-Collins syndrome.

## DISCUSSION

This study aimed to provide a comprehensive overview of the phenotypic spectrum of Miller syndrome and improve differentiation from related facial dysostosis syndromes. A total of 44 cases were analyzed.

Our findings revealed significant phenotypic variability in Miller syndrome, comparable to Treacher-Collins and Nager syndrome cases. Nevertheless, craniofacial and limb anomalies were consistently reported in all cases of Miller syndrome. Comparisons with Treacher-Collins and Nager syndrome patients highlighted differences in the frequency of several features, including extracraniofacial anomalies, orofacial clefts, and anomalies of the external ears and eyelids.

### Strengths and Limitations

To better understand this study’s findings, it is crucial to acknowledge its limitations. Firstly, there was a scarcity of genetically confirmed cases of Miller syndrome, including only 8 out of 44 cases (18.2%). This can partially be explained by the historical context, as the causative gene was only identified in 2010,^2,3^ and 68.2% (N=30) of the cases were described before this discovery. However, genetic confirmation was also not obtained or reported for 42.9% (N=6) of the cases described after 2010.^35,39,41^ The lack of genetic diagnoses raises the possibility that some included cases may represent other (acrofacial dysostosis) syndromes, potentially influencing the observed prevalence of specific features. To address this, future studies, including case reports, should prioritize genetic confirmation. For previously published clinical cases, reanalysis using Sanger sequencing, targeted next-generation sequencing, or more advanced techniques such as whole-genome sequencing or RNA sequencing may be considered. This would not only support research on causative genes and enhance diagnostic accuracy but also ensure that the studied condition truly represents Miller syndrome.

In addition, the study’s reliance on a systematic literature review carries inherent limitations. Although this approach provides high specificity (ie, a reported characteristic can be reasonably assumed to be present), it lacks sensitivity (ie, the absence of a reported characteristic does not confirm it is absent). Consequently, characteristics with high reported prevalence, such as orofacial clefts and cupped ears, are likely the most reliable, whereas those with lower prevalence should be interpreted with caution, including eyelid anomalies, hearing function, and extracraniofacial anomalies.

Despite these limitations, our study represents the most robust examination of Miller syndrome to date, encompassing 44 cases. Our results offer clinicians and researchers valuable insights into the phenotypic spectrum of Miller syndrome for better recognition and distinction with other facial dysostosis syndromes. For instance, the term “postaxial acrofacial dysostosis” seems less fitting given the thumb (ie, preaxial) anomalies reported in some cases. Moreover, these results offer critical information for counseling parents of unborn children with a prenatal diagnosis of Miller syndrome. Overall, a detailed understanding of the phenotype not only enhances our knowledge of the condition but also establishes a foundation for further research and improvements in the care of affected individuals.

### Future Direction

To facilitate research for rare conditions such as Miller syndrome in the future, collaborative efforts among clinicians and researchers should be encouraged, for instance, by establishing centralized databases. In Europe, the ERN CRANIO (European Reference Network for rare and/or complex craniofacial anomalies and ear, nose, and throat disorders) is currently working on this for facial dysostosis syndromes. Databases for craniosynostoses, cleft lip and palate, and rare genetic deafness have already been established. Such databases facilitate the collection and analysis of larger and more diverse data sets, enabling more robust research and improvement of care for rare conditions.

To make a jumpstart for this collaborative research on Miller syndrome, 19 specialized centers within the ERN CRANIO across multiple countries were contacted. Although 2 new cases of Miller syndrome were identified, both individuals declined to participate due to concerns about maintaining their anonymity in light of this condition’s extreme rarity. Thus, centralized registries may even be a prerequisite for identifying enough cases while ensuring patient anonymity in publications.

Nevertheless, given the extreme rarity, case reports and small case series will likely continue to be the primary means of disseminating knowledge about the syndrome. To improve the quality of future research contributions, comprehensive reporting of all observed characteristics is recommended, including the conscientious exclusion of anomalies, rather than solely reporting what is present. In line with this, a detailed checklist for phenotypic evaluation in Miller syndrome cases is provided in our study (Supplemental Digital File 3, Supplemental Digital Content 3, http://links.lww.com/SCS/H874).

Another important step in research on this rare condition would involve not only the collection of patient characteristics but also documenting the treatment pathways. Publishing treatment experiences for Miller syndrome could lay the groundwork for developing future treatment recommendations and providing direction for clinicians and patients. Although standardized treatment protocols for Miller syndrome are infeasible due to its extreme rarity and phenotypic variability, the ERN CRANIO currently provides guidance through its clinical consensus statement for facial dysostosis syndromes.^47^ In addition, the ERN CRANIO has implemented the Clinical Patient Management System (CPMS), a secure web-based platform that enables clinicians to collaborate virtually and share information across institutions. Accessible to ERN CRANIO centers, affiliated partners, and non-ERN centers through guest access, the CPMS ensures that patients with rare craniofacial conditions, including Miller syndrome, receive care informed by the collective expertise and the latest knowledge available.

In conclusion, this study provides a comprehensive review of the phenotypic spectrum of Miller syndrome, a rare condition within the group of facial dysostosis syndromes. Despite the limitations of limited genetic confirmation and reliance on case reports, our findings offer valuable insights for clinicians, researchers, and future parents. To advance research and care for rare conditions like Miller syndrome, future efforts should prioritize genetic confirmation, establish international research collaborations, and encourage comprehensive reporting through our proposed checklist. This will not only enhance our knowledge but also improve diagnostic accuracy and care for patients with Miller syndrome.

## Supplementary Material

SUPPLEMENTARY MATERIAL
