# Supplementary material for: Unveiling the Phenotypic Spectrum of Miller Syndrome: A Systematic Review
Source: J Craniofac Surg. 2025 May 19;36(8):e1243–7. doi: 10.1097/SCS.0000000000011501 (PMC12537037; doi:10.1097/SCS.0000000000011501)
Supplement: SUPPLEMENTARY MATERIAL [file scs-36-e1243-s002.docx]

**Supplementary Digital Content 2 – Search strings**

| **Database searched** | **Platform** | **Years of coverage** | **Records** | **Records after duplicates removed** |
| --- | --- | --- | --- | --- |
| Medline ALL | Ovid | 1946 - Present | 103 | 103 |
| Embase | Embase.com | 1971 - Present | 142 | 52 |
| Web of Science Core Collection* | Web of Knowledge | 1975 - Present | 117 | 26 |
| CINAHL | EBSCO | 1982 - Present | 15 | 11 |
| **Total** | | | **377** | **192** |

*Science Citation Index Expanded (1975-present) ; Social Sciences Citation Index (1975-present) ; Arts & Humanities Citation Index (1975-present) ; Conference Proceedings Citation Index- Science (1990-present) ; Conference Proceedings Citation Index- Social Science & Humanities (1990-present) ; Emerging Sources Citation Index (2005-present)

No other database limits were used than those specified in the search strategies

**Medline**

(Mandibulofacial Dysostosis / AND Micrognathism / AND Limb Deformities, Congenital /) OR Acrofacial dysostosis, Nager type.nm. OR (Miller-syndrome* OR Miller-s-syndrome* OR ((Postaxial* OR Post-axial* OR Genee* OR Wiedemann* OR Miller*) AND (Acrofacial* OR Acro-facial*) ADJ3 Dysostos*) OR POADS OR (Wildervanck ADJ3 Smith)).mp.

**Embase**

('mandibulofacial dysostosis'/de AND micrognathia/de AND 'limb malformation'/de) OR (Miller-syndrome* OR Miller-s-syndrome* OR ((Postaxial* OR Post-axial* OR Genee* OR Wiedemann* OR Miller*) AND (Acrofacial* OR Acro-facial*) NEAR/3 Dysostos*) OR POADS OR (Wildervanck NEAR/3 Smith)):ab,ti,kw,de

**Web of science**

TS=((Miller-syndrome* OR Miller-s-syndrome* OR ((Postaxial* OR Post-axial* OR Genee* OR Wiedemann* OR Miller*) AND (Acrofacial* OR Acro-facial*) NEAR/2 Dysostos*) OR POADS OR (Wildervanck NEAR/2 Smith)))

**CINAHL**

(MH Mandibulofacial Dysostosis AND MH Micrognathism AND MH Limb Deformities, Congenital) OR TI(Miller-syndrome* OR Miller-s-syndrome* OR ((Postaxial* OR Post-axial* OR Genee* OR Wiedemann* OR Miller*) AND (Acrofacial* OR Acro-facial*) N2 Dysostos*) OR POADS OR (Wildervanck N2 Smith)) OR AB(Miller-syndrome* OR Miller-s-syndrome* OR ((Postaxial* OR Post-axial* OR Genee* OR Wiedemann* OR Miller*) AND (Acrofacial* OR Acro-facial*) N2 Dysostos*) OR POADS OR (Wildervanck N2 Smith))
