# Supplementary material for: Unveiling the Phenotypic Spectrum of Miller Syndrome: A Systematic Review
Source: J Craniofac Surg. 2025 May 19;36(8):e1243–7. doi: 10.1097/SCS.0000000000011501 (PMC12537037; doi:10.1097/SCS.0000000000011501)
Supplement: SUPPLEMENTARY MATERIAL [file scs-36-e1243-s003.docx]

**SDC 3 - Checklist for Phenotypical Evaluation of Miller Syndrome**

The following checklist is developed for phenotypical evaluations of patients with a clinical or genetic diagnosis of Miller syndrome, as well as for patients with a high suspicion of Miller syndrome. Characteristics are grouped into larger categories (i.e., eyes and eyelids, limbs, etc.). If no anomalies are identified within a category, one can specify this at the beginning of the section and skip to the next section.

Firstly, please specify the basis of the Miller syndrome diagnosis by checking one of the following boxes:

Genetically confirmed diagnosis

Clinical diagnosis

High suspicion

In case of a genetically confirmed diagnosis, please specify the identified gene mutation:

|  |
| --- |

Next, please specify the baseline characteristics:

Sex:  Male  Female

| Age at evaluation |  | **Not Determined** |
| --- | --- | --- |
| Pregnancy duration (weeks) |  |  |
| Birth weight (kilograms) |  |  |
| Birth length (centimeters) |  |  |
| Occipitofrontal circumference (centimeters) |  |  |
| Family history |  |  |
| Other diagnoses |  |  |

| Physical development |  |
| --- | --- |
| Cognitive development |  |
| Psychomotor development |  |

Please specify the presence of hearing loss and, if so, the amount of hearing loss in dB.

Conductive hearing loss:  Present (R       dB; L       dB)  Absent  Not Determined
Sensorineural hearing loss:  Present (R       dB; L       dB)  Absent  Not Determined

| **Anomalies of the:** | **Present** | **Absent** | **Not Determined** | **Comments** |
| --- | --- | --- | --- | --- |
| **Eyelids and Lacrimal system** |  |  |  |  |
| Absent or scarce eyelashes |  |  |  |  |
| Blepharophimosis |  |  |  |  |
| Downslanting palpebral fissures |  |  |  |  |
| Ectropion |  |  |  |  |
| Epicanthus |  |  |  |  |
| Lacrimal duct anomalies |  |  |  |  |
| Lower eyelid coloboma(s) |  |  |  |  |
| Upper eyelid coloboma(s) |  |  |  |  |
| Upslanting palpebral fissures |  |  |  |  |
| Other anomalies |  | | | |
|  |  |  |  |  |
| **Eyes and orbitae** |  |  |  |  |
| (Pseudo-)strabismus |  |  |  |  |
| Corneal anomalies |  |  |  |  |
| Enophthalmos |  |  |  |  |
| Exophthalmos |  |  |  |  |
| Hypertelorism |  |  |  |  |
| Peri-orbital bony clefts |  |  |  |  |
| Refractive errors |  |  |  |  |
| Other anomalies |  | | | |
|  |  |  |  |  |
| **Nose** |  |  |  |  |
| Anteverted nostrils |  |  |  |  |
| Choanal atresia |  |  |  |  |
| Depressed nasal bridge |  |  |  |  |
| Other anomalies |  | | | |
|  |  |  |  |  |
| **Craniofacial hypoplasia** |  |  |  |  |
| Malar hypoplasia |  |  |  |  |
| Maxillary hypoplasia |  |  |  |  |
| Mandibular hypoplasia (micrognathia) |  |  |  |  |
| Other anomalies |  | | | |
|  |  |  |  |  |
| **Orofacial cleft** |  |  |  |  |
| Bilateral cleft lip, alveolus, and palate |  |  |  |  |
| Unilateral cleft lip, alveolus, and palate |  |  |  |  |
| Unilateral cleft lip and alveolus |  |  |  |  |
| Unilateral cleft lip |  |  |  |  |
| Cleft palate |  |  |  |  |
| Other: |  | | | |
|  |  |  |  |  |
| **Remaining mouth anomalies** |  |  |  |  |
| Ankyloglossia |  |  |  |  |
| Conical teeth |  |  |  |  |
| Glossoptosis |  |  |  |  |
| Long philtrum |  |  |  |  |
| Macrostomia |  |  |  |  |
| Microstomia |  |  |  |  |
| Other anomalies |  | | | |
|  |  |  |  |  |
| **Ears** |  |  |  |  |
| Adherent lobuli |  |  |  |  |
| Cupped ear deformity |  |  |  |  |
| Low set ears |  |  |  |  |
| Middle ear hypoplasia/aplasia |  |  |  |  |
| Narrow ear canals |  |  |  |  |
| Pre-auricular tags |  |  |  |  |
| Other anomalies |  | | | |
|  |  |  |  |  |
| **Hands** |  |  |  |  |
| Abnormal 1^st^ digital ray(s) |  |  |  |  |
| Abnormal 2^nd^-4^th^ digital ray(s) |  |  |  |  |
| Abnormal 5^th^ digital ray(s) |  |  |  |  |
| Abnormal metacarpal(s) |  |  |  |  |
| Absent 1^st^ digital ray(s) |  |  |  |  |
| Absent 2^nd^-4^th^ digital ray(s) |  |  |  |  |
| Absent 5^th^ digital ray(s) |  |  |  |  |
| Absent metacarpal(s) |  |  |  |  |
| Brachydactyly |  |  |  |  |
| Camptodactyly |  |  |  |  |
| Carpal fusion(s) |  |  |  |  |
| Clinodactyly |  |  |  |  |
| Single palmar crease(s) |  |  |  |  |
| Syndactyly |  |  |  |  |
| Other anomalies |  | | | |
|  |  |  |  |  |
| **Forearms** |  |  |  |  |
| Ulna dysplasia/hypoplasia |  |  |  |  |
| Absent ulna |  |  |  |  |
| Radius dysplasia/hypoplasia |  |  |  |  |
| Absent radius |  |  |  |  |
| Radioulnar synostosis |  |  |  |  |
| Other anomalies |  | | | |
|  |  |  |  |  |
| **Feet** |  |  |  |  |
| Abnormal 2^nd^-4^th^ ray |  |  |  |  |
| Abnormal 5^th^ ray |  |  |  |  |
| Absent 2^nd^-4^th^ ray |  |  |  |  |
| Absent 5^th^ ray |  |  |  |  |
| Absent lateral cuneiform bone(s) |  |  |  |  |
| Brachyctyly |  |  |  |  |
| Syndactyly |  |  |  |  |
| Other anomalies |  | | | |
|  |  |  |  |  |
| **Genital anomalies** |  |  |  |  |
| *(For male cases)* |  |  |  |  |
| Cryptorchidism |  |  |  |  |
| Micropenis |  |  |  |  |
| Phimosis |  |  |  |  |
| Other anomalies |  | | | |
|  |  |  |  |  |
| **Remaining anomalies of the neck, trunk, and pelvis** |  |  |  |  |
| Coccygeal sinus |  |  |  |  |
| Congenital dermal melanocytosis (Slate grey nevus) |  |  |  |  |
| Dysplastic nipples |  |  |  |  |
| Hypoplastic pectoral muscle |  |  |  |  |
| Inguinal hernia |  |  |  |  |
| Periareolar skin tags |  |  |  |  |
| Polythelia (accessory nipple(s)) |  |  |  |  |
| Webbed neck |  |  |  |  |
| Other anomalies |  | | | |
|  |  |  |  |  |
| **Vertebral anomalies** |  |  |  |  |
| Abnormal segmentation of the sternum |  |  |  |  |
| Brachycephaly |  |  |  |  |
| Cervical rib(s) |  |  |  |  |
| Fusion of spinous process(es) |  |  |  |  |
| Hypoplastic spinous process(es) |  |  |  |  |
| Pectus excavatum |  |  |  |  |
| Plagiocephaly |  |  |  |  |
| Platyspondyly |  |  |  |  |
| Scoliosis |  |  |  |  |
| Other anomalies |  | | | |
|  |  |  |  |  |
|  |  |  |  |  |
| **Cardiac anomalies** |  |  |  |  |
| Atrial septal defect (ASD) |  |  |  |  |
| Patent ductus arteriosus |  |  |  |  |
| Patent foramen ovale |  |  |  |  |
| Ventricular septal defect (VSD) |  |  |  |  |
| Other anomalies |  | | | |
|  |  |  |  |  |
| **Central nervous system anomalies** |  |  |  |  |
| Agenesis of corpus callosum |  |  |  |  |
| Anencephaly |  |  |  |  |
| Cerebral atrophy |  |  |  |  |
| Microcephaly |  |  |  |  |
| Other anomalies |  | | | |
|  |  |  |  |  |
| **Renal anomalies** |  |  |  |  |
| Hydronephrosis |  |  |  |  |
| Renal hypoplasia |  |  |  |  |
| Other anomalies |  | | | |
|  |  |  |  |  |
| **Gastrointestinal anomalies** |  |  |  |  |
| Anal prolapse |  |  |  |  |
| Cholestasis |  |  |  |  |
| Duodenum malrotation |  |  |  |  |
| Liver fibrosis |  |  |  |  |
| Pyloric stenosis |  |  |  |  |
| Other anomalies |  | | | |

| **Remaining anomalies** |  |
| --- | --- |
