# Supplementary material for: Unveiling the Phenotypic Spectrum of Miller Syndrome: A Systematic Review
Source: J Craniofac Surg. 2025 May 19;36(8):e1243–7. doi: 10.1097/SCS.0000000000011501 (PMC12537037; doi:10.1097/SCS.0000000000011501)
Supplement: SUPPLEMENTARY MATERIAL [file scs-36-e1243-s004.docx]

Supplemental Digital Table 1 – Case Overview

| **Fam. #** | **Case #** | **Sex** | **Diagnosis** | **Country of origin** | **Quality** | **Article** | **Year** | **Ref.** |
| --- | --- | --- | --- | --- | --- | --- | --- | --- |
| 1 | 1 | F | Genetic | Austria | Average | Al Kaissi et al. | 2011 | ^18^ |
| 2 | 2 | NA | Clinical | Italy | Low | Barbuti et al. | 1989 | ^19^ |
| 3 | 3 | M | Genetic | Poland | Average | Bukowska-Olech et al. | 2020 | ^20^ |
| 4 | 4 | F | Clinical | Poland, Belgium | Low | Chrzanowska et al. | 1989 | ^8^ |
| 5 | 5 | M | Clinical |  |  |  |  |  |
|  | 6 | M | Clinical |  | Low* | Chrzanowska & Fryns | 1993 | ^21^ |
|  | 7 | M | Clinical |  |  |  |  |  |
| 6 | 8 | F | Clinical | United-Kingdom | Low | Donnai et al. | 1987 | ^13^ |
| 7 | 9 | M | Clinical |  |  |  |  |  |
| 8 | 10 | F | Clinical |  |  |  |  |  |
| 9 | 11 | M | Genetic | Australia | High | Duley et al. | 2016 | ^22^ |
| 10 | 12 | M | Clinical | USA | Low | Fineman | 1981 | ^23^ |
|  | 13 | F | Clinical |  | Average | Miller et al. | 1979 | ^24^ |
| 11 | 14 | M | Clinical | Belgium | Average | Fryns & Van den Berghe | 1988 | ^25^ |
| 12 | 15 | M | Clinical | Italy | Low | Giannotti et al. | 1992 | ^26^ |
|  | 16 | F | Clinical |  |  |  |  |  |
| 13 | 17 | M | Clinical | Germany | Low* | Hauss-Albert & Passarge | 1988 | ^27^ |
| 14 | 18 | F | Genetic | Japan | High | Kinoshita et al. | 2011 | ^28^ |
| 15 | 19 | M | Clinical | USA | Average | Miller et al. | 1979 | ^24^ |
| 16 | 20 | M | Clinical |  |  |  |  |  |
| 17 | 21 | M | Genetic | Norway | High | Mero et al. | 2024 | ^29^ |
| 18 | 22 | M | Clinical | Former Yugoslavia | Low | Neumann et al. | 1996 | ^30^ |
|  | 23 | F | Clinical |  |  |  |  |  |
| 19 | 24 | F | Clinical | New Zealand | Average | Ogilvy-Stuart & Parsons | 1991 | ^31^ |
|  | 25 | M | Clinical |  |  |  |  |  |
| 20 | 26 | M | Clinical | USA | Low | Opitz & Stickler | 1987 | ^32^ |
| 21 | 27 | F | Clinical | Brazil | Low | Pereira et al. | 1992 | ^33^ |
| 22 | 28 | F | Clinical |  |  |  |  |  |
|  | 29 | M | Clinical |  | Low | Richieri-Costa & Guion-Almeida | 1989 | ^34^ |
| 23 | 30 | M | Genetic | United-Kingdom, Germany, Spain | Average | Rainger et al. | 2012 | ^35^ |
| 24 | 31 | F | Genetic |  |  |  |  |  |
| 25 | 32 | F | Genetic |  |  |  |  |  |
| 26 | 33 | F | Clinical |  |  |  |  |  |
| 27 | 34 | M | Clinical |  |  |  |  |  |
| 28 | 35 | M | Clinical |  |  |  |  |  |
| 29 | 36 | F | Clinical |  |  |  |  |  |
| 30 | 37 | F | Clinical | New Zealand | Average | Richards | 1987 | ^36^ |
| 31 | 38 | M | Clinical | USA | Low* | Robinow et al. | 1986,  1990 | ^37,38^ |
|  | 39 |  |  |  |  |  |  |  |
|  | 40 |  |  |  |  |  |  |  |
| 32 | 41 | M | Clinical | Egypt | Average | Shawky et al. | 2014 | ^39^ |
| 33 | 42 | F | Clinical | USA | Low | Stevenson et al. | 1991 | ^40^ |
| 34 | 43 | M | Clinical | India | Average | Urs et al | 2014 | ^41^ |
| 35 | 44 | M | Clinical | France | Low | Vigneron et al. | 1991 | ^42^ |

*Joanna Briggs Institute checklist for Case Report was applied, despite article being a letter

Supplemental Digital Table 2A – Phenotypical characteristics in patients with clinical or genetic diagnosis of Miller syndrome (N = 44)

|  | **Clinical diagnosis (n=36)** | | | **Genetic diagnosis (n=8)** | | |
| --- | --- | --- | --- | --- | --- | --- |
| **Characteristic** | **Yes** | **No** | **NR** | **Yes** | **No** | **NR** |
| **General** | - | - | - | - | - | - |
| Normal cognition | 18 (50.0%) | 3 (8.3%) | 15 (41.7%) | 6 (75.0%) | 2 (25.0%) | 0 (0%) |
| Hearing loss | 5 (13.9%) | 8 (22.2%) | 23 (63.9%) | 3 (37.5%) | 2 (25.0%) | 3 (37.5%) |
| Vertebral Anomalies | 10 (27.8%) | 2 (5.6%) | 24 (66.7%) | 2 (25.0%) | 0 (0%) | 6 (75.0%) |
| Cardiac Anomalies | 3 (8.3%) | 12 (33.3%) | 21 (58.3%) | 4 (50.0%) | 3 (37.5%) | 1 (12.5%) |
| CNS Anomalies | 3 (8.3%) | 3 (8.3%) | 30 (83.3%) | 1 (12.5%) | 1 (12.5%) | 6 (75.0%) |
| Gastrointestinal Anomalies | 2 (5.6%) | 6 (16.7%) | 28 (77.8%) | 2 (25.0%) | 2 (25.0%) | 4 (50.0%) |
| Renal Anomalies | 1 (2.8%) | 3 (8.3%) | 32 (88.9%) | 1 (12.5%) | 0 (0%) | 7 (87.5%) |
|  |  |  |  |  |  |  |
| **Eyelid Anomalies** | **25 (69.4%)** | **0 (0%)** | **11 (30.6%)** | **6 (75.0%)** | **0 (0%)** | **2 (25.0%)** |
| Slanting palpebral fissures | 15 (41.7%) | 1 (2.8%) | 20 (55.6%) | 4 (50.0%) | 0 (0%) | 4 (50.0%) |
| *Down-slanting* | 10 (27.8%) | - | - | 3 (37.5%) | - | - |
| *Up-slanting* | 5 (13.9%) | - | - | 1 (11.1%) | - | - |
| Ectropion | 13 (36.1%) | 3 (8.3%) | 20 (55.6%) | 1 (12.5%) | 0 (0%) | 7 (87.5%) |
| Eyelid coloboma | 7 (19.4%) | 11 (30.6%) | 18 (50.0%) | 2 (25.0%) | 2 (25.0%) | 4 (50.0%) |
| *Lower eyelid(s)* | 4 (11.1%) | - | - | 2 (22.2%) | - | - |
| *Upper eyelid(s)* | 3 (8.3%) | - | - | 0 (0%) | - | - |
| Blepharophimosis | 7 (19.4%) | 1 (2.8%) | 28 (77.8%) | 1 (12.5%) | 0 (0%) | 7 (87.5%) |
| Absent/scarce lower eyelashes | 5 (13.9%) | 2 (5.6%) | 32 (74.4%) | 3 (37.5%) | 1 (12.5%) | 4 (50.0%) |
|  |  |  |  |  |  |  |
| **Orofacial Cleft** | **28 (77.8%)** | **3 (8.3%)** | **5 (13.9%)** | **6 (75.0%)** | **2 (25.0%)** | **0 (0%)** |
| Isolated cleft palate | 24 (66.7%) | 7 (19.4%) | 5 (13.9%) | 5 (62.5%) | 3 (37.5%) | 0 (0%) |
| *Complete* | 15 (41.7%) | - | - | 4 (50.0%) | - | - |
| *Soft palate only* | 8 (22.2%) | - | - | 0 (0%) | - | - |
| *Submucous* | 1 (2.8%) | - | - | 1 (12.5%) | - | - |
| Cleft lip and palate | 4 (11.1%) | 27 (75.0%) | 5 (13.9%) | 1 (12.5%) | 7 (87.5%) | 0 (0%) |
|  |  |  |  |  |  |  |
| **Other Mouth Anomalies** | **11 (30.6%)** | **0 (0%)** | **25 (69.4%)** | **3 (37.5%)** | **0 (0%)** | **5 (62.5%)** |
| Long philtrum | 5 (13.9%) | 0 (0%) | 31 (86.1%) | 3 (37.5%) | 4 (50.0%) | 1 (12.5%) |
| Glossoptosis | 4 (11.1%) | 0 (0%) | 32 (88.9%) | 0 (0%) | 0 (0%) | 8 (100%) |
| Microstomia | 2 (5.6%) | 3 (8.3%) | 30 (83.3%) | 2 (25.0%) | 0 (0%) | 6 (75.0%) |
| Conical teeth | 3 (8.3%) | 1 (2.8%) | 32 (88.9%) | 1 (12.5%) | 0 (0%) | 7 (78.5%) |
|  |  |  |  |  |  |  |
| **Craniofacial Hypoplasia** | **32 (88.9%)** | **1 (2.8%)** | **3 (8.3%)** | **7 (87.5%)** | **0 (0%)** | **1 (12.5%)** |
| Micrognathia | 29 (80.6%) | 4 (11.1%) | 3 (8.3%) | 4 (50.0%) | 0 (0%) | 4 (50.0%) |
| Malar, maxillary, or midface hypoplasia | 26 (72.2%) | 1 (2.8%) | 9 (25.0%) | 6 (75.0%) | 0 (0%) | 2 (25.0%) |
|  |  |  |  |  |  |  |
| **External Ear Anomalies** | **21 (58.3%)** | **8 (22.2%)** | **7 (19.4%)** | **7 (87.5%)** | **1 (12.5%)** | **0 (0%)** |
| Cupped ear(s) | 14 (38.9%) | 15 (41.7%) | 7 (19.4%) | 6 (75.0%) | 2 (25.0%) | 0 (0%) |
| Low-set ear(s) | 9 (25.0%) | 4 (11.1%) | 23 (63.9%) | 4 (50.0%) | 0 (0%) | 4 (50.0%) |
|  |  |  |  |  |  |  |

NR = Not reported; CNS = Central Nervous System

Supplemental Digital Table 2B – Phenotypical characteristics in patients with clinical or genetic diagnosis of Miller syndrome (N = 44)

|  | **Clinical diagnosis (n=36)** | | | **Genetic diagnosis (n=8)** | | |
| --- | --- | --- | --- | --- | --- | --- |
| **Characteristic** | **Yes** | **No** | **NR** | **Yes** | **No** | **NR** |
| **Hand Anomalies** | **34 (94.4%)** | **2 (5.6%)** | **0 (0%)** | **8 (100%)** | **0 (0%)** | **0 (0%)** |
| Absent or malformed 5^th^ ray(s) | 32 (88.9%) | 4 (11.1%) | 0 (0%) | 7 (87.5%) | 1 (12.5%) | 0 (0%) |
| Absent or malformed thumb(s) | 16 (44.4%) | 13 (36.1%) | 7 (19.4%) | 1 (12.5%) | 7 (87.5%) | 0 (0%) |
| Syndactyly | 8 (22.2%) | 18 (50.0%) | 10 (27.8%) | 3 (37.5%) | 4 (50.0%) | 1 (12.5%) |
| Camptodactyly | 5 (13.9%) | 11 (30.6%) | 20 (55.6%) | 2 (25.0%) | 4 (50.0%) | 2 (25.0%) |
| Single palmar crease | 3 (8.3%) | 0 (0%) | 33 (91.7%) | 2 (25.0%) | 0 (0%) | 6 (75.0%) |
| Carpal fusion | 3 (8.3%) | 11 (30.6%) | 22 (61.1%) | 1 (12.5%) | 0 (0%) | 7 (87.5%) |
|  |  |  |  |  |  |  |
| **Forearm Anomalies** | **18 (50.0%)** | **11 (30.6%)** | **7 (19.4%)** | **5 (62.5%)** | **2 (25.0%)** | **1 (12.5%)** |
| Radio-ulnar synostosis | 6 (16.7%) | 17 (47.2%) | 13 (36.1%) | 1 (12.5%) | 2 (25.0%) | 5 (62.5%) |
|  |  |  |  |  |  |  |
| **Foot Anomalies** | **33 (91.7%)** | **2 (5.6%)** | **1 (2.8%)** | **7 (87.5%)** | **1 (12.5%)** | **0 (0%)** |
| Absent or malformed 5^th^ ray(s) | 30 (83.3%) | 4 (11.1%) | 2 (5.6%) | 6 (75.0%) | 2 (25.0%) | 0 (0%) |
| Syndactyly | 5 (13.9%) | 13 (36.1%) | 18 (50.0%) | 3 (37.5%) | 4 (50.0%) | 1 (12.5%) |
|  |  |  |  |  |  |  |
| **Genital Anomalies** | **7 (19.4%)** | **2 (5.6%)** | **27 (75.0%)** | **0 (0%)** | **1 (12.5%)** | **7 (78.5%)** |
| Cryptorchidism* | 6 (25.0%) | 5 (20.8%) | 13 (54.2%) | 0 (0%) | 1 (33.3%) | 2 (66.7%) |
| Small penis or micro-penis* | 4 (16.7%) | 2 (8.3%) | 18 (75.0%) | 0 (0%) | 0 (0%) | 3 (100%) |
|  |  |  |  |  |  |  |
| **Remaining Anomalies** | - | - | - | - | - | - |
| Polythelia | 7 (19.4%) | 5 (13.9%) | 24 (66.7%) | 3 (37.5%) | 0 (0%) | 5 (62.5%) |
| Pectus excavatum | 4 (11.1%) | 1 (2.8%) | 31 (86.1%) | 6 (75.0%) | 1 (12.5%) | 1 (12.5%) |
| Middle ear hypoplasia | 4 (11.1%) | 3 (8.3%) | 30 (83.3%) | 0 (0%) | 1 (12.5%) | 7 (87.5%) |
| Choanal atresia | 4 (11.1%) | 8 (22.2%) | 24 (66.7%) | 0 (0%) | 3 (37.5%) | 5 (62.5%) |

NR = Not reported; *Percentages calculated in males only

Supplemental Digital Table 3 – Phenotypical characteristics in genetically confirmed facial dysostosis patients. Percentages reflect only cases where the presence or absence of characteristics was reported, with the number of cases indicated in brackets.

| **Syndrome** | **Treacher Collins** | | | **Nager** | **Miller** |
| --- | --- | --- | --- | --- | --- |
| **Affected gene** | **TCOF1** | **POLR1D** | **POLR1C** | **SF3B4** | **DHODH** |
| Reference(s) | ^43-45,49^ | ^43-45^ | ^46^ | ^6,47^ | This study |
| Total number of reported patients | 114 | 27 | 3 | 9 | 8 |
|  |  |  |  |  |  |
| **General** |  |  |  |  |  |
| Hearing loss | 90.5% (95) | 100% (5) | 100% (3) | 71.4% (7) | 60.0% (5) |
| Vertebral Anomalies | NA | 14.3% (7) | NA | NA | 100% (2) |
| Cardiac Anomalies | 7.0% (114) | 0% (27) | NA | 33.3% (9) | 57.1% (7) |
| CNS Anomalies | NA | NA | NA | NA | 50.0% (2) |
| Gastrointestinal Anomalies | 1.8% (114) | 0% (27) | NA | NA | 50.0% (4) |
| Renal Anomalies | 4.3% (46) | 0% (4) | NA | 33.3% (3) | 100% (1) |
| Genital anomalies | NA | NA | NA | 20.0% (5) | 0% (1) |
|  |  |  |  |  |  |
| **Eyelid Anomalies** | 100% (97) | 85.7% (7) | 66.7% (3) | 85.7% (7) | 100% (6) |
| Down-slanting palpebral fissures | 100% (97) | 85.7% (7) | 66.7% (3) | 88.9% (9) | 75.0% (4) |
| Lower eyelid coloboma | 60.9% (92) | 42.9% (7) | 66.7% (3) | 0% (4) | 50.0% (4) |
| Absent/scarce lower eyelashes | NA | NA | NA | 57.1% (7) | 75.0% (4) |
|  |  |  |  |  |  |
| **Orofacial cleft** | 25.3% (91) | 16.7% (6) | 50% (2) | 77.8% (9) | 75.0% (8) |
| Cleft palate | 25.3% (91) | 16.7% (6) | 50% (2) | 77.8% (9) | 75.0% (8) |
| Cleft lip | NA | NA | NA | 0% (6) | 12.5% (8) |
|  |  |  |  |  |  |
| **Craniofacial hypoplasia** | 99.0% (98) | 100% (7) | 100% (3) | 100% (9) | 100% (7) |
| Midface, malar, or maxillary hypoplasia | 99.0% (98) | 85.7% (7) | 100% (3) | 100% (9) | 100% (6) |
| Micrognathia | 87.8% (98) | 100% (7) | 100% (3) | 100% (9) | 100% (4) |
|  |  |  |  |  |  |
| **External ear anomalies** | 73.7% (99) | 57.1% (7) | 100% (3) | 88.9% (9) | 87.5% (8) |
|  |  |  |  |  |  |
| **Hand anomalies** | 1.4% (71) | 0% (7) | NA | 100% (9) | 100% (8) |
| Absent or malformed 5^th^ ray | 0% (71) | 0% (7) | NA | 0% (2) | 87.5 % (8) |
| Absent or malformed thumbs | 0% (71) | 0% (7) | NA | 100% (9) | 12.5% (8) |
| Syndactyly | 0% (71) | 0% (7) | NA | 37.5% (8) | 42.9% (7) |
|  |  |  |  |  |  |
| **Forearm anomalies** | 0% (71) | 0% (7) | NA | 62.5% (8) | 71.4% (7) |
| Radio-ulnar synostosis | 0% (71) | 0% (7) | NA | 28.6% (7) | 33.3% (3) |
|  |  |  |  |  |  |
| **Foot anomalies** | 0% (71) | 0% (7) | NA | 100% (6) | 87.5% (8) |
|  |  |  |  |  |  |
| **Remaining anomalies** |  |  |  |  |  |
| Choanal atresia | 18.5% (81) | 0% (7) | 0% (1) | 0% (9) | 0% (3) |
| Microcephaly | 3.3% (60) | 0% (6) | NA | 0% (9) | 0% (2) |
|  |  |  |  |  |  |

NA: Data not available

Supplemental Digital Table 4 – Reported mutations on the DHODH gene in Miller syndrome cases

| **Case #** | **HGVS coding (cDNA)** | **HGVS protein (p.)** | **Genomic location** | **Inheritance origin** |
| --- | --- | --- | --- | --- |
| 1 | c.403C>T | p.(Arg135Cys)^1^ | Exon 3 | Paternal |
|  | c.1036C>T | p.(Arg346Trp)^2^ | Exon 8 | Maternal |
| 3 | c.128C>T | p.(Pro43Leu) | Exon 2 | Maternal |
|  | c.403C>T | p.(Arg135Cys)^1^ | Exon 3 | Paternal |
| 11 | c.165-166delCA | p.(His55Glufs*21) | Exon 2 | Maternal |
|  | c.925C>T | p.(Arg309Trp) | Exon 7 | Paternal |
| 18 | c.82T>C | p.(Leu28Pro) | Exon 2 | Maternal |
|  | c.1040G>A | p.(Ala347Thr) | Exon 8 | Paternal |
| 21 | c.403C > T | p.(Arg135Cys)^1^ | Exon 3 | NA |
|  | c.829G > A | p.(Asp277Asn) | Exon 7 | NA |
| 30 | c.404C>T | p.(Arg135Cys)^1^ | Exon 3 | NA |
|  | c.1036C>T | p.(Arg346Trp)^2^ | Exon 8 | NA |
| 31 | c.404C>T | p.(Arg135Cys)^1^ | Exon 3 | NA |
|  | c.1175A>G | p.(Asp392Gly) | Exon 9 | NA |
| 32 | c.1069G>A | p.(Ala357Thr) | Exon 8 | NA |
|  | c.1022C>T | p.(Arg326Ter) | Exon 8 | NA |

^1,2^protein changes that were reported more than once
